# Human health effects of traffic-related air pollution (TRAP): a scoping review protocol

**DOI:** 10.1186/s13643-019-1106-5

**Published:** 2019-08-29

**Authors:** Carlyn J. Matz, Marika Egyed, Robyn Hocking, Shayesta Seenundun, Nick Charman, Nigel Edmonds

**Affiliations:** 1 Air Health Effects Assessment Division, Health Canada, 269 Laurier Ave W, Ottawa, ON K1A 0K9 Canada; 2 Information/Knowledge Management Division, Health Canada, 200 Eglantine Driveway, Ottawa, ON K1A 0K9 Canada

**Keywords:** Traffic-related air pollution (TRAP), Epidemiology, Human health, Scoping review

## Abstract

**Background:**

Traffic-related air pollution (TRAP) is one of the major sources of exposure in urban areas and has been associated with a wide range of adverse human health effects. Much of the Canadian population is regularly exposed to TRAP as a result of daily activities (e.g., commuting) and a significant portion of the population resides in close proximity to major roadways. The objective of this scoping review is to develop an evidence map of the epidemiological literature of the human health effects of exposure to TRAP, to support future reviews and assessments by Health Canada.

**Methods:**

Literature searches will be conducted in Ovid EMBASE and Ovid MEDLINE database. DistillerSR will be used to manage the review process. Two reviewers will independently screen the studies in a two-part process (title and abstract; full text) for eligibility. Epidemiological studies and reviews will be included if they report on the human health effects of exposure to TRAP. Data collection will include study design parameters and human health outcomes evaluated in the study. A descriptive analysis will be used to provide a high-level summary of the number of studies evaluating the different types of health effects and cross-tabulations by study design parameters.

**Discussion:**

The scoping review will be used to identify subject areas for more detailed review and evaluation of the human health effects of TRAP by the Air Health Effects Assessment Division of Health Canada.

**Electronic supplementary material:**

The online version of this article (10.1186/s13643-019-1106-5) contains supplementary material, which is available to authorized users.

## Background

In urban areas, traffic is one of the major sources of air pollutants. The mixture of vehicle exhausts, secondary pollutants formed in the atmosphere, evaporative emissions from vehicles, and non-combustion emissions (e.g., road dust, tire wear) is referred to as traffic-related air pollution (TRAP). Exposure to this complex and variable mixture of gasses and particles has been linked to a range of health effects from adverse birth outcomes to dementia (e.g., [1, 2]). In 2010, the Health Effects Institute published a critical review of the literature on emissions, exposure, and health effects of TRAP, identifying associations between TRAP exposure and adverse cardiorespiratory effects including exacerbation of asthma, incident asthma, reduced lung function, myocardial infarction, progression of atherosclerosis, and cardiovascular mortality [3]. Since the publication of the Health Effects Institute critical review, the scientific literature regarding the human health effects of TRAP has increased, as TRAP remains an active area of research in environmental epidemiology.

The Air Health Effects Assessment Division of Health Canada conducts human health risk assessments of air pollutants, such as diesel exhaust [4] and nitrogen dioxide [5] with the goal of supporting programs and policies dedicated to improving air quality for Canadians. Health Canada maintains an active interest in TRAP as an air pollution source for multiple reasons. Firstly, health effects associated with TRAP exposure have been extensively reported on in the scientific literature, given the importance of air pollution as an environmental health issue and the importance of traffic as a source of air pollutants. Furthermore, Canadians spend approximately 4–7% of daily time in microenvironments influenced by moderate to heavy traffic, such as traveling in a vehicle or being engaged in active transportation [6]. Additionally, in Canada, it has been estimated that ten million people (about 32% of the population) live within 500 m of highways or 100 m of major urban roads [7].

Given the anticipated large body of literature in this field, the aim of this scoping review by Health Canada is to develop an evidence map of the human health effects associated with TRAP based on epidemiological studies and reviews. This evidence map will be the primary outcome of the scoping review. To accomplish this aim, the scoping review will employ systematic review techniques, incorporating inclusion and exclusion criteria developed by the Health Effects Institute [3], to identify TRAP-specific studies. The evidence map will be used to support secondary outcomes of the scoping review which are to efficiently identify, prioritize, and select areas for further, more in-depth review and assessment by Health Canada.

## Methods

### Scoping review aim

The aim is to conduct a scoping review of the epidemiological literature of the human health effects of exposure to TRAP. This scoping review will characterize the available studies in order to map the evidence available based on study design and adverse health outcomes. The evidence maps (or charts) generated will facilitate identification and selection of subject areas for further review and evaluation by the Air Health Effects Division of Health Canada as well as identify areas of knowledge gaps for future research. The framework for the scoping review includes:

1. Identifying the research question;
2. Identifying relevant studies and search strategy;
3. Study selection;
4. Charting the data;
5. Collating, summarizing, and reporting the results.

### Research questions

The primary research question for this scoping review is as follows: What is the current body of scientific literature regarding the association between TRAP exposure and adverse human health endpoints (including effects in various systems: respiratory, cardiovascular, immunological, reproductive/developmental, and nervous, as well as other health endpoints such as cancer and mortality)? The research sub-questions include:

1. What is the current body of scientific literature pertaining to the health effects of TRAP exposure with respect to:
  - Different life stages [i.e., infants (< 1 year), children and adolescents (< 18 years), adults (18–55 years), and seniors (55+ years)]?
  - Susceptible populations [i.e., people with pre-existing diseases (diabetes, asthma, COPD, or heart disease)]?
  - Sex-based analysis (i.e., males only, females only)?
  - Ambient exposure-based studies (i.e., general population) and occupational exposure-based studies (i.e., limited to occupation groups such as traffic wardens, bus drivers, taxi drivers)?
  - Type of article (i.e., primary research studies, systematic reviews, critical reviews, and meta-analyses)?

### Identifying the relevant studies and search strategy

#### Types of studies

The scoping review will include primary epidemiological research articles and some review types (as described below) published in peer-reviewed journals that address the scoping review objectives. The following observational study designs will be included: case-control, cohort, cross-sectional, panel (including randomized, crossover design), ecological, time-series, and case-crossover. Experimental studies will be considered only if human subjects were involved in the study (i.e., controlled human exposure studies). Review types that will be considered include the following: systematic reviews, meta-analyses, scoping reviews, and critical reviews. Conference proceedings and narrative reviews will be excluded.

#### Participants

Studies of humans of all ages, sexes, health status, and geographic locations will be included. Risk assessments and health impact analyses will be excluded. Studies in animal models, cell culture, or in vitro will be excluded.

#### Exposure

For the scoping review, the TRAP and traffic exposure metrics considered for inclusion or exclusion will be adapted from those used by the Health Effects Institute’s critical review of TRAP [3], as described below:

Inclusion criteria (must meet at least 1 of the following):

1. Measures based on distance or length
  Maximum distance to nearest main road or highway
  Distance to street canyons
  Length of main streets within buffer zones around homes or schools
2. Measures of traffic density
  Density on nearest road
  Average density estimated from road networks within buffer zones around homes or school
  Note of street canyons with buffers
3. Modeling
  Dispersion modeling of traffic exposure
  Other modeling techniques for estimating of traffic exposure (e.g., a land-use regression model)
  Traffic-specific source apportionment
4. Subjects in occupations characterized by exposure to traffic (e.g., taxi drivers, truckers, traffic wardens)
5. Pollutant surrogates of traffic exposure such as NO_2_, CO, elemental carbon (or black smoke), or other (e.g., benzene, diesel exhaust, lead) included as a criterion in the absence of a specific metric, if the monitors or other sources of measurement used to estimate the pollutant-exposure surrogate could reasonably be related to traffic (e.g., roadway-specific monitoring or subjects living within short distances of fixed monitors)
6. Subjects in locations characterized by exposure to traffic (e.g., high vs. low traffic exposure sites)

Exclusion criteria (exclude if one or more of the following are met):

1. Studies in which traffic exposure was based solely on subject self-reporting, without other more direct or objective measures of exposure
2. Occupational studies in which exposure could not be specifically linked to traffic sources of vehicle or engine exhaust (e.g., miners, railroad workers, construction workers), as this exposure is different from that of the general public
3. Occupational studies in which exposure was assessed as diesel and/or gasoline exhaust without specifying traffic as the source, as these could include non-traffic related sources of exhaust and this exposure is different from that of the general public

#### Outcomes

There will be no exclusions or restrictions on health effects, as the scoping review will include all human health outcomes. All-cause and cause-specific mortalities will be included. For morbidity, clinical effects, sub-clinical effects, and biomarkers will be included and organized by the following sub-headings: respiratory, cardiovascular, immunological, reproductive/developmental, and neurological. Full details of the health outcomes considered for charting are provided in Additional file 1. These health effect sub-headings were chosen based on those utilized in previous Health Canada health risk assessments of air pollutants [4, 5]. Studies based on measures of health care utilization (e.g., hospital admissions and doctor visits) will also be included. There will be no restrictions on outcomes based on the type of risk estimate reported.

### Search strategy

A literature search will be conducted by the Health Canada Library, from the Ovid EMBASE and Ovid MEDLINE databases. An additional search for relevant articles will be conducted from the reference lists of identified review studies. The full search strategies are presented in Additional file 2. All database searches will be restricted to 2000–present and English or French language. Publications from 2000 to present will be considered in order to have a partial overlap with the Health Effects Institute’s critical review [3], which considered 1980–2008. Additionally, this represents almost the last 20 years of research on the health effects of TRAP, which has gained much interest during this time frame.

#### Study selection

Following deduplication, the database literature search results will be uploaded into DistillerSR (Evidence Partners, Ottawa, Canada), an online systematic review manager. Two reviewers will independently screen titles and abstracts from the databases for eligibility. In the next step, two reviewers will independently review and evaluate the full-text articles. Reviewers will identify the publication type (e.g., primary research study or review article). Primary research articles will be evaluated to confirm inclusion based on exposure assessment criteria. Eligible review articles will be evaluated for the inclusion of TRAP-specific studies. For these two screening steps, small batches of records will be used for form testing within DistillerSR and reviewer calibration. During the screening process, any conflicts will be resolved between screeners and a third party, if necessary. A PRISMA Flow Diagram will be generated using DistillerSR once all the included articles have been identified.

#### Charting the data

Data extraction forms in DistillerSR will be used to guide data collection from the included studies to map the evidence. Primary research articles will be charted by study design parameters and health effect endpoints. For each study, one reviewer will identify the following: type of exposure (ambient air or occupational-based), life stage(s) of study participants (all ages, infants, children, youth, adults, seniors), sex of study participants (male, female, both), and health status of study participants (healthy or pre-existing disease such as asthma, chronic obstructive pulmonary disease, diabetes, or heart disease). Studies will also be charted for health effect endpoints evaluated including the following: mortality, respiratory effects, cardiovascular effects, immunological effects, reproductive and developmental effects, neurological effects, genotoxicity and cancer, and other endpoints (reviewers will be able to specify endpoint). Each of these broad health effect categories will have several sub-groupings to provide additional details on the endpoints evaluated in each study. Eligible review articles will be similarly charted and classified by type of review (systematic review, scoping review, critical review, or meta-analysis).

The data collection step will include a quality assurance/quality control step applied to approximately 15% of studies to verify the accuracy and completeness of the data collection process. This quality assurance/quality control step will be performed independently by a different reviewer than the one who conducted the data collection.

#### Collating, summarizing, and reporting the results

Descriptive tables will be developed to summarize the number (i.e., count) of primary studies and reviews that have evaluated the different health effects, by broad category (e.g., respiratory health effects) and sub-groupings (e.g., respiratory mortality, asthma, lung function). Additionally, health effects will be cross-tabulated with study design parameters (e.g., life stages, susceptible populations, etc). These tables will be used to identify literature-rich subject areas as well as those more poorly characterized. The Air Health Effects Assessment Division of Health Canada will use the summarized results to identify subject areas for future review and evaluation. A completed copy of the PRISMA-ScR checklist is included as an additional file (Additional file 3).

#### Quality assessment

The purpose of this scoping review is to identify all the available evidence on the association between human health effects and TRAP. As such, study quality or a formal risk of bias will not be assessed, nor will it be used as a basis for exclusion of studies, in keeping with scoping review guidelines [8].

## Discussion

This scoping review will generate an evidence map of the epidemiological literature of the human health effects of TRAP. Due to increasing interest in and methodological developments for the evaluation of the health effects of air pollution and TRAP, a large number of studies is anticipated to meet the search criteria. The literature search strategy was developed by a Health Canada librarian in collaboration with the project coordinator. This process was performed to refine the search strategy to the extent possible, given the high-level and broad nature of this scoping review. The screening steps will be used to identify TRAP-specific studies from the larger body of air pollution health effects research. For future phases of this project, once subject areas for review and evaluation are identified from the scoping review, quality evaluations will be performed on the subset of relevant studies, including risk of bias or review quality appraisals.

Although the search strategy was clearly defined, some limitations in this scoping review are anticipated. Studies may be omitted if they are not indexed in the databases searched or if they are not available in English or French.

Overall, the scoping review will be used to generate an evidence map to efficiently identify, prioritize, and select subject areas for more in-depth review and evaluation by Health Canada. The subsequent reports and evaluations that will be derived from this scoping review may be used to support policies and programs and development of communication and outreach materials regarding TRAP and the associated health effects, as per key objectives of Health Canada’s program on air quality and health.

## Additional files

Additional file 1: Health outcomes. (DOCX 15 kb)

Additional file 2: Literature search strategy. (DOCX 19 kb)

Additional file 3: PRISMA-ScR checklist. (PDF 645 kb)

## Data Availability

Further information related to this review can be provided upon reasonable request. Interested readers should contact the corresponding author.

## Abbreviations

TRAP: Traffic-related air pollution
