# Supplementary material for: Human health effects of traffic-related air pollution (TRAP): a scoping review protocol
Source: Syst Rev. 2019 Aug 29;8:223. doi: 10.1186/s13643-019-1106-5 (PMC6714303; doi:10.1186/s13643-019-1106-5)
Supplement: Supplementary file 1 — Health outcomes. (DOCX 15 kb) [file 13643_2019_1106_MOESM1_ESM.docx]

**Additional file 1**

| **Main Health Outcome Grouping** | **Subgrouping** |
| --- | --- |
| All-cause mortality |  |
| Respiratory Effects | o Mortality  o Asthma (e.g. medication use, asthma wheeze, airway resistance)  o Symptoms (e.g. cough, wheeze not attributed to asthma, eye/nose/throat irritation)  o Hospitalization and ER Visits  o Lung function (e.g. spirometry, FEV, FVC)  o Inflammation and oxidative stress (e.g. FeNO)  o Other (to be identified by reviewer) |
| Cardiovascular Effects | o Mortality  o Heart rate variability (e.g. RMSSD, SDNN)  o Cardiac function (e.g. EKG measures)  o Blood pressure  o Systemic inflammation  o Hospitalization and ER visits  o Morbidities (e.g. atherosclerosis, coronary artery disease, ischemic heart disease, myocardial infarction, arrhythmia, cardiac arrest)  o Other (to be identified by reviewer) |
| Immunological Effects | o Mortality  o Heart rate variability (e.g. RMSSD, SDNN)  o Cardiac function (e.g. EKG measures)  o Blood pressure  o Systemic inflammation  o Hospitalization and ER visits  o Morbidities (e.g. atherosclerosis, coronary artery disease, ischemic heart disease, myocardial infarction, arrhythmia, cardiac arrest)  o Other (to be identified by reviewer) |
| Reproductive and Developmental Effects | o Fertility, reproduction, pregnancy (e.g. sperm effects, live births)  o Birth outcomes (e.g. effects identified in newborns or infants; pre-term birth, size at birth)  o Post-natal development (e.g. developmental effects in children, including neurodevelopmental)  o Other (to be identified by reviewer) |
| Neurological Effects | o Neuro-function in adults (e.g. memory, response times)  o Neurodevelopmental effects in children (e.g. Autism, ADHD/ADD, behavioural)  o Parkinson’s disease  o Alzheimer’s disease  o Mental health (e.g. depression, anxiety)  o Other (to be identified by reviewer) |
| Genotoxicity and Cancer | o Childhood cancers   - Leukemia and Lymphoma - Other cancers   o Adult cancers   - Lung cancer - Leukemia and Lymphoma - Other cancers   o Molecular markers of genotoxicity (e.g. 8-OHdG) |
| Other diseases, morbidities, or health / well-being status | o Diabetes (includes Types 1 and 2)  o Obesity (e.g. BMI, adiposity)  o Overall health or well-being (typically self-reported)  o Endocrine / hormone (e.g. thyroid hormomes)  o Cardiometabolic syndrome  o Osteoporosis and related (e.g. bone mineral density)  o Epigenetic changes (e.g. DNA methylation)  o Eye or ocular related  o Rheumatoid arthritis  o Crohn’s disease, colitis, inflammatory bowel disease  o Mammographic density and other breast tissue measures  o Sleeping disorders  o Aging  o Progression of physical disability  o Hepatoxicity  o Other mortalities  o Other (to be identified by reviewer) |
