# Supplementary material for: Human health effects of traffic-related air pollution (TRAP): a scoping review protocol
Source: Syst Rev. 2019 Aug 29;8:223. doi: 10.1186/s13643-019-1106-5 (PMC6714303; doi:10.1186/s13643-019-1106-5)
Supplement: Supplementary file 2 — Literature search strategy. (DOCX 19 kb) [file 13643_2019_1106_MOESM2_ESM.docx]

**Additional file 2**

**A. Ovid Embase (1974 to Date TBD)**

| 1. exhaust gas/ |
| --- |
| 2. exp motor vehicle/ |
| 3. car driving/ |
| 4. exp traffic/ |
| 5. "traffic and transport"/ |
| 6. nitrogen oxide/ |
| 7. nitrogen dioxide/ |
| 8. carbon monoxide/ |
| 9. carbon dioxide/ |
| 10. ozone/ |
| 11. volatile organic compound/ |
| 12. black carbon/ |
| 13. particulate matter/ |
| 14. exp polycyclic aromatic hydrocarbon/ |
| 15. exp air pollution/ |
| 16. ((distanc* or proximit* or close*) adj3 (traffic* or road* or highway* or transitway*)).tw,kw. |
| 17. traffic exposure*.tw,kw. |
| 18. fume/ |
| 19. ((automobile* or automotive* or autocar* or autobus* or motor* or vehic* or taxi* or diesel* or gasoline* or engine? or car or cars or truck* or bus or buses or bussing or highway* or high way* or motorway* or motor way or road* or parkade* or parking* or carpark* or car park* or traffic*) adj4 (exhaust? or emission* or pollut* or vapor* or vapour* or volatile* or effluvia* or smoke* or fume? or haze? or smog* or nitrogen oxide* or NOx or 11104-93-1 or carbon monoxide* or 630-08-0 or carbon dioxide or 124-38-9 or volatile organic compounds or VOC or VOCs or Peroxyacetyl nitrate or 2278-22-0 or polycyclic aromatic hydrocarbon* or arene* or PAH or polyaromatic hydrocarbon* or polynuclear aromatic hydrocarbon*)).tw,kw. |
| 20. 1 or ((or/2-5) and (or/6-18)) or 19 [TRAP] |
| 21. exp health/ |
| 22. public health/ |
| 23. "physical disease by etiology and pathogenesis"/ or acute disease/ or exp aplasia/ or exp ascites/ or exp atrophy/ or exp bleeding/ or exp calcification/ or exp channelopathy/ or chemically induced disorder/ or exp chronic disease/ or exp complication/ or critical illness/ or exp cyst/ or exp deformity/ or exp degeneration/ or exp diverticulosis/ or exp dysplasia/ or exp dystrophy/ or exp ectopic tissue/ or exp edema/ or exp effusion/ or exp emphysema/ or endemic disease/ or environmental disease/ or epidemic/ or exp fibrosis/ or exp fistula/ or exp "genetic and familial disorders"/ or exp healing impairment/ or exp hernia/ or exp hyperplasia/ or exp hypertrophy/ or exp hypoplasia/ or exp hypotrophy/ or exp iatrogenic disease/ or idiopathic disease/ or exp infection/ or exp inflammation/ or exp ischemia/ or exp "lesions and defects"/ or exp malnutrition/ or exp metaplasia/ or exp necrosis/ or neglected disease/ or neointima/ or exp neoplasm/ or exp "neovascularization (pathology)"/ or non communicable disease/ or exp occupational disease/ or pandemic/ or exp pseudotumor/ or rare disease/ or recurrent disease/ or relapse/ or reversal reaction/ or exp sclerosis/ or exp "stenosis, occlusion and obstruction"/ or exp stone formation/ or exp storage disease/ or exp swelling/ or syndrome/ or systemic disease/ or terminal disease/ or exp thromboembolism/ or exp torsion/ or exp "toxicity and intoxication"/ or exp ulcer/ |
| 24. exp mental disease/ |
| 25. physical disease/ or exp physical disease by anatomical structure/ or exp physical disease by body function/ or exp "physical disease by composition of body fluids, excreta and secretions"/ or exp physical disease by developmental age/ |
| 26. diseases/ |
| 27. exp mortality/ |
| 28. mortality risk/ |
| 29. exp epidemiology/ |
| 30. exp epidemiological monitoring/ |
| 31. exp epidemiological data/ |
| 32. environmental health/ |
| 33. genotoxicity/ |
| 34. genetic damage/ |
| 35. mutagenic activity/ |
| 36. mutagenicity/ |
| 37. exp postnatal development/ |
| 38. exp toxicity/ |
| 39. exp biological functions/ |
| 40. environmental health/ |
| 41. environmental stress/ |
| 42. "quality of life"/ |
| 43. hospitalization/ or hospital admission/ |
| 44. (IQR or interquartile range* or inter quartile range*).tw,kw. |
| 45. ((population* or human* or citizen* or nation* or public or communit* or individual* or people* or person* or man or men or woman* or women* or child* or infan* or toddler* or newborn* or neonat* or baby or babies or adolecen* or teenage* or preteen* or preadolescen* or premenarch* or pre menarch* or adult* or elderly or seniors) adj3 (health* or disease*)).tw,kw. |
| 46. or/21-45 [Health Effects] |
| 47. limit 46 to ((english or french) and yr="2000 -Current") |
| 48. 20 and 47 [TRAP + Human Health] |
| 49. limit 48 to human |
| 50. (human* or person* or people* or man or men? or wom?n or child* or infan* or toddler* or newborn* or neonat* or baby or babies or adolecen* or teenage* or preteen* or preadolescen* or premenarch* or pre menarch* or adult* or elderly or seniors).tw,kw. |
| 51. exp human/ |
| 52. 48 and (50 or 51) |
| 53. nonhuman/ |
| 54. 48 not 53 |
| 55. 49 or 52 or 54 |
| 56. limit 55 to conference abstract status |
| 57. 55 not 56 |

**B. Ovid Medline®  Epub Ahead of Print, In-Process & Other Non-Indexed Citations, Ovid MEDLINE® Daily, Ovid MEDLINE and Versions® (1946 to Date TBD)**

| 1. Vehicle Emissions/ |
| --- |
| 2. exp motor vehicles/ |
| 3. transportation/ |
| 4. Automobile Driving/ |
| 5. Parking Facilities/ |
| 6. exp Nitrogen Oxides/ |
| 7. Carbon Monoxide/ |
| 8. Carbon Dioxide/ |
| 9. Ozone/ |
| 10. Volatile Organic Compounds/ |
| 11. Soot/ |
| 12. particulate matter/ or exp dust/ or smog/ |
| 13. exp Polycyclic Aromatic Hydrocarbons/ |
| 14. air pollution/ or air pollution, indoor/ |
| 15. ((distanc* or proximit* or close*) adj3 (traffic* or road* or highway* or transitway*)).tw,kf. |
| 16. traffic exposure*.tw,kf. |
| 17. ((automobile* or automotive* or autocar* or autobus* or motor* or vehic* or taxi* or diesel* or gasoline* or engine? or car or cars or truck* or bus or buses or bussing or highway* or high way* or motorway* or motor way or road* or parkade* or parking* or carpark* or car park* or traffic*) adj4 (exhaust? or emission* or pollut* or vapor* or vapour* or volatile* or effluvia* or smoke* or fume? or haze? or smog* or nitrogen oxide* or NOx or 11104-93-1 or carbon monoxide* or 630-08-0 or carbon dioxide or 124-38-9 or volatile organic compounds or VOC or VOCs or Peroxyacetyl nitrate or 2278-22-0 or polycyclic aromatic hydrocarbon* or arene* or PAH or polyaromatic hydrocarbon* or polynuclear aromatic hydrocarbon*)).tw,kf. |
| 18. 1 or ((or/2-5) and (or/6-16)) or 17 [TRAP] |
| 19. exp Health/ |
| 20. exp "diseases (non mesh)"/ |
| 21. exp Mental Disorders/ |
| 22. exp morbidity/ |
| 23. exp mortality/ |
| 24. exp Epidemiology/ |
| 25. Epidemiological Monitoring/ |
| 26. exp "growth and development"/ |
| 27. "Quality of Life"/ |
| 28. exp Hospitalization/ |
| 29. (IQR or interquartile range* or inter quartile range*).tw,kf. |
| 30. (health* or disease* or illness* or mortalit* or morbidit* or disorder* or sick*).tw,kf. |
| 31. or/19-30 [Health Effects] |
| 32. limit 31 to ((english or french) and yr="2000 -Current") |
| 33. 18 and 32 [TRAP + Human Health] |
| 34. limit 33 to human |
| 35. (human* or person* or people* or man or men? or wom?n or child* or infan* or toddler* or newborn* or neonat* or baby or babies or adolecen* or teenage* or preteen* or preadolescen* or premenarch* or pre menarch* or adult* or elderly or seniors).tw,kf. |
| 36. exp human/ |
| 37. 33 and (35 or 36) |
| 38. exp models animal/ |
| 39. exp animal experimentation/ |
| 40. 33 not (37 or 38) |
| 41. 50 or 53 or 40 |
